# Stability Analysis and Design of *n*-DOF Vibration Systems Containing Both Semi-Active and Passive Mechanical Controllers

**DOI:** 10.3390/s24051600

**Published:** 2024-02-29

**Authors:** Kai Wang, Wei Xu

**Affiliations:** Key Laboratory of Advanced Process Control for Light Industry (Ministry of Education), School of Internet of Things Engineering, Jiangnan University, Wuxi 214122, China; 6211905050@stu.jiangnan.edu.cn

**Keywords:** vibration control, stability analysis, switched system, semi-active inerter, network synthesis

## Abstract

This paper is concerned with the stability analysis and design of the *n*-DOF (*n*-degree-of-freedom) mass-chain vibration systems containing both semi-active and passive mechanical controllers. Based on Lyapunov’s stability theory, sufficient conditions are derived for the *n*-DOF vibration system containing a semi-active switched inerter and a passive mechanical network with the first-order admittance to be globally asymptotically stable. Furthermore, the optimization designs of a quarter-car vibration control system and a three-storey building vibration system are conducted together with the derived stability results, and the instability cases contradicting the stability conditions are presented for illustration. The optimization and simulation results show that the combination of semi-active and passive mechanical controllers in vibration systems can clearly enhance system performances in comparison with the conventional semi-active or passive control. The novelty of this paper is that the stability problem of a general *n*-DOF vibration system that simultaneously contains a semi-active controller and a first-order passive controller is investigated for the first time, where such a system combines the advantages of both semi-active and passive mechanical controllers. The investigations and results can provide an essential foundation for further exploring the stability problems of more general systems, and can be applied to the controller designs of many vibration systems in practice.

## 1. Introduction

With the invention of inerters [1,2,3], it is possible to systematically realize any passive mechanical system as the physical interconnection of dampers, springs, inerters, etc., which has motivated the recent investigations on the synthesis of passive networks under low-complexity constraints [4,5,6,7,8,9]. Passive mechanical networks containing dampers, springs, and inerters (or called damper–spring–inerter networks) have been widely applied as passive mechanical controllers to many vibration control systems, such as seat suspension systems [10], beam-type vibration systems [11], vehicle suspension systems [12,13,14,15,16], vibration absorbers [17,18], bridge vibration systems [19], wind turbine systems [20], storage tanks [21,22], building vibration systems [23], etc. The results have shown that the low-complexity mechanical networks containing inerters can always provide better system performances compared with the conventional damper–spring networks. The vibration control systems employing passive mechanical controllers have the advantages of high reliability, a low cost, being energy-saving, etc., and the design process based on network synthesis can be divided into two steps [24,25]. The first step is to determine the transfer function (such as admittance, impedance, etc.) of a suitable passive controller to meet the requirements of asymptotic stability, system performances, etc. The second step is to apply the theory of passive network synthesis to physically realize the transfer function of the passive controller as a low-complexity damper–spring–inerter network.

**Figure 1:**
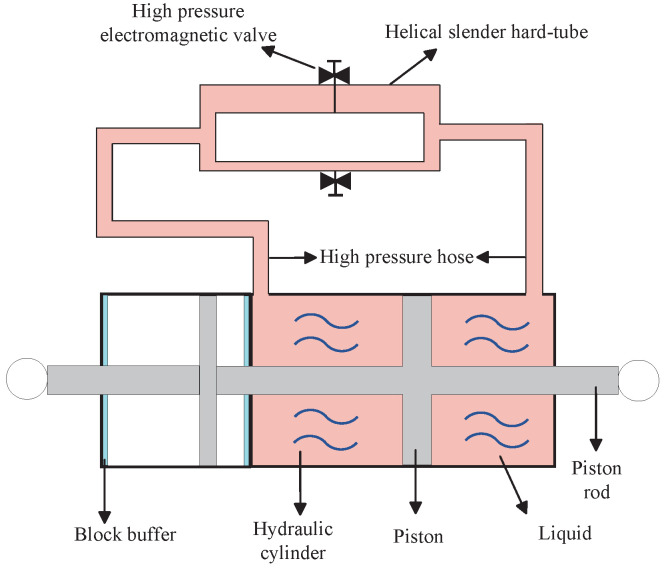
The prototype of a semi-active fluid inerter [28].

On the other hand, the semi-active mechanical control making use of semi-active elements has been widely applied to many vibration systems to improve performances [26,27,28,29], where parameters of semi-active elements are adjustable by proper parameter control laws. Compared with the active control methods, the semi-active mechanical control always consumes less energy [24]. In addition to semi-active dampers and semi-active springs, semi-active inerters have been proposed, such as the semi-active fluid inerters [28] (see Figure 1) and the controllable-inertia-flywheel-based ball-screw inerters [29]. The inertance of the semi-active fluid inerter can be adjusted by controlling the two high-pressure electromagnetic valves.

As a specific class of semi-active control, switching control [30,31,32,33], which has only two or several control modes, is much easier to implement than continuous semi-active control. Recently, switched inerters have been applied to many vibration control systems, so that the system performances can be enhanced [26,27,28,34,35]. However, introducing switched mechanical elements may lead to system instability [36,37,38,39]. Therefore, the stability problems of semi-active switched vibration systems need to be investigated for the optimization of designs. For instance, Corless and Leitmann [36] derived an instability condition of an SDOF system containing a semi-active switched spring. In [37], a series of instability results for *n*-DOF vibration systems containing *n* semi-active inerters have been given (including the results for SDOF systems when *n* = 1). In [38], necessary and sufficient conditions for the global asymptotic stability of an SDOF system containing at most three semi-active switched elements were derived, and a series of global asymptotic stability results for the *n*-DOF (n≥1) vibration system containing a semi-active inerter were derived. Ramaratnam and Jalili [39] derived the stability condition of an SDOF system containing a semi-active switched spring by the Lyapunov approach.

In recent years, many investigations have focused on the control problems of *n*-DOF mass-chain vibration systems [20,40,41,42], such as isolators, dynamic vibration absorbers, vehicle suspension systems, wind turbine systems, multi-storey building vibration systems, etc. For instance, Yamamoto [40] studied the synthesis problem of an *n*-DOF vibration system whose adjacent masses are interconnected by passive damper–spring–inerter networks. Li and Chen [41] investigated the achievable dynamic responses of an *n*-DOF undamped vibration system containing an active controller. Hu et al. [42] were concerned with the inherent stability problem of an *n*-DOF vibration control system containing continuously adjustable semi-active inerters. In [20], the relationship between the three parts of the *n*-DOF vibration systems, which are main devices, auxiliary devices, and mechanical networks, was investigated.

**Figure 2:**
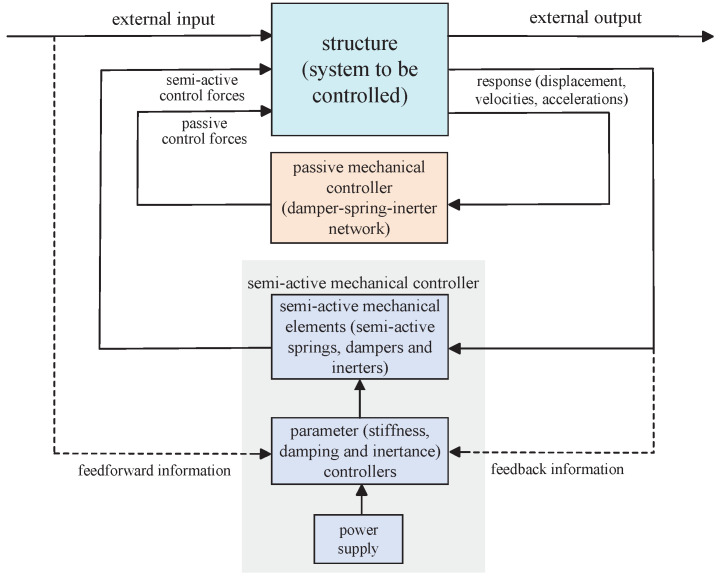
The block diagram of vibration control systems containing semi-active and passive mechanical controllers.

Together with the discussions as above, the vibration control systems that simultaneously apply the semi-active and passive mechanical controllers are expected to provide better system performances and to maintain the advantages of high reliability, low cost, etc. (see [43]). The block diagram of such a vibration control system is shown in Figure 2, where the semi-active mechanical controllers are implemented by semi-active mechanical elements whose parameters can be adjusted, and the passive mechanical controllers are implemented by damper–spring–inerter networks whose admittance is a low-order positive-real function. The asymptotic stability analysis of the *n*-DOF vibration systems simultaneously containing semi-active and passive mechanical controllers has seldom been investigated and needs to be explored, which can provide essential foundations for control system designs.

This paper is dedicated to investigating the global asymptotic stability problem of a general *n*-DOF mass-chain vibration system that contains both semi-active and passive mechanical controllers, and applies the derived stability results to the designs of two vibration control systems, where the semi-active mechanical controller is the switched inerter satisfying the relative-velocity–relative-acceleration switching law (see [27,28]), and the passive mechanical controller is the one-port damper–spring–inerter network whose admittance is any first-order positive-real function. Based on the Lyapunov approach, a sufficient condition for the global asymptotic stability of the general *n*-DOF vibration systems is derived in Proposition 1, where the semi-active and passive mechanical controllers can be installed between any two adjacent masses. Moreover, a sufficient condition for the global asymptotic stability of a specific *n*-DOF vibration system is presented in Proposition 2. Then, the stability results are applied to the optimization designs of a quarter-car suspension control system and a three-storey building vibration system. The simulation results show that using both semi-active and passive mechanical controllers in vibration systems can clearly improve system performances compared with the conventional semi-active or passive control approach. Compared with the previous related investigations in [38], this paper introduces the passive mechanical network whose admittance is any first-order positive-real function.

In this paper, the stability problem of a general *n*-DOF vibration system that simultaneously contains a semi-active controller and a first-order passive controller is investigated for the first time and such a system combines the advantages of both semi-active and passive mechanical controllers. General stability results for *n*-DOF vibration systems are derived, which can be applied to the optimization designs of many practical vibration systems, such as suspension systems, building vibrations, wind turbine vibrations, etc. The investigations and results of this paper can provide an essential foundation for further exploring the stability problems and designs of more general vibration control systems.

The remaining part of this paper is organized as follows. Section 2 presents the preliminaries of this paper. The model formulation is presented in Section 3. The stability results of *n*-DOF vibration systems are derived in Section 4. Section 5 applies the stability conditions derived in Section 4 to the optimization designs of a quarter-car suspension control system and a three-storey building vibration control system. Conclusions are made in Section 6.

## 2. Preliminaries

**Figure 3:**
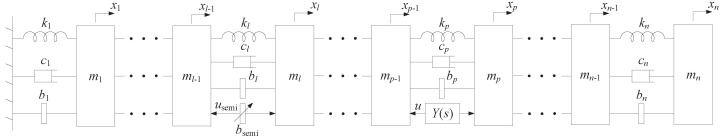
The *n*-DOF vibration control system containing one semi-active mechanical controller and one passive mechanical controller, where $m_{i}>0$, $k_{i}>0$, $c_{i}\geq 0$, $b_{i}\geq 0$ for i∈{1,2,…,n}, the switched inerter $b_{\mathrm{semi}}$ is the semi-active mechanical controller installed between $m_{l-1}$ and $m_{l}$ for a certain l∈{1,2,…,n}, and Y(s) is the admittance of the passive mechanical controller installed between $m_{p-1}$ and $m_{p}$ for a certain p∈{1,2,…,n} (*l* and *p* are independent of each other).

This paper aims to investigate the Lyapunov stability problem of the *n*-DOF vibration system shown in Figure 3, where *n* is the number of degrees of freedom (n≥1), and to apply the stability results to the optimization designs of a suspension control system (n=2) and a three-storey building vibration system (n=3), respectively. In this section, we will present some preliminary definitions and lemmas of passive network synthesis and Lyapunov’s stability theory to be utilized in the investigations of this paper.

The following Definitions 1–3 present some basic concepts about passive mechanical networks.

**Figure 4:**
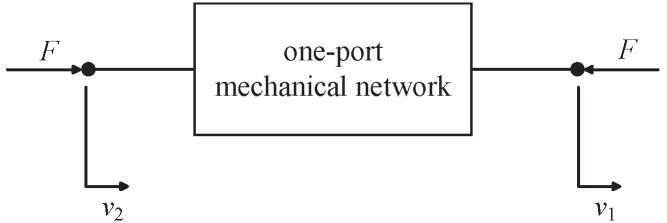
The one-port mechanical network, where *F* is the force and $v=v_{2}-v_{1}$ is the relative velocity of two terminals [2].

**Definition** **1** ([8])**.** *A one-port mechanical network (see Figure 4) is defined as being passive, if there exists K∈R such that the external force F and relative velocity v of the two terminals satisfy*

$$-\int_{t_{0}}^{t_{1}}F\left(t\right)v\left(t\right)dt<K$$

*for all $t_{1}\geq t_{0}$.*

**Definition** **2** ([5,25])**.** *For any real-rational function H(s), it is defined as being positive real, if H(s) is analytic and satisfies ℜ(H(s))≥0 for all ℜ(s)>0, where ℜ(·) denotes the real part of ·∈C.*

**Definition** **3** ([1,2])**.** *The admittance (resp. impedance) of a one-port linear time-invariant mechanical network is defined as being $Y\left(s\right)=\hat{F}/\hat{v}$ (resp. $Z\left(s\right)=\hat{v}/\hat{F}$), where $\hat{F}$ and $\hat{v}$ represent the Laplace transforms of the external force F and relative velocity v of the two terminals, respectively.*

Then, the following Lemmas 1 and 2 provide some fundamental conclusions of network synthesis, which will be applied in Section 5.1 and Section 5.2.

**Lemma** **1** ([2,25])**.** *The admittance Y(s) (resp. impedance Z(s)) of any one-port linear time-invariant passive mechanical network is positive real, and any positive-real admittance Y(s) (resp. impedance Z(s)) is realizable by a one-port linear time-invariant passive mechanical network only consisting of a finite number of dampers, springs, and inerters (called one-port damper–spring–inerter network for brevity).*

For any positive-real admittance Y(s) (resp. impedance Z(s)), we can choose a suitable passive network synthesis procedure, such as the Bott–Duffin synthesis procedure [44,45], to realize Y(s) (resp. Z(s)) as a one-port damper–spring–inerter network (see [1,8] for details).

**Lemma** **2** ([25])**.** *Any first-order real-rational admittance Y(s) in the form of*

(1) $$Y\left(s\right)=\frac{\alpha_{1}s+\alpha_{0}}{\beta_{1}s+\beta_{0}},$$

**Figure 5:**
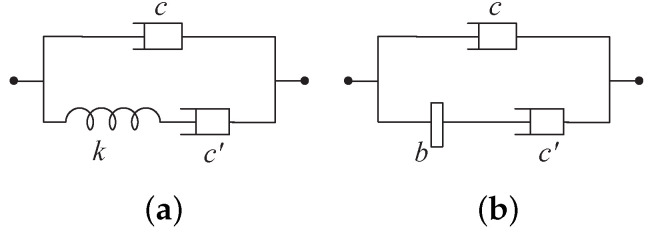
The one-port three-element passive mechanical network realizations of the first-order positive-real admittance Y(s) in (1): (**a**) $\alpha_{1}\beta_{0}-\alpha_{0}\beta_{1}\leq 0$; (**b**) $\alpha_{1}\beta_{0}-\alpha_{0}\beta_{1}>0$.

*where $\alpha_{1}\geq 0$, $\alpha_{0}>0$, $\beta_{1}>0$, and $\beta_{0}>0$, is a positive-real function, and is realizable by one of the three-element networks in Figure 5. Specifically, if $\alpha_{1}\beta_{0}-\alpha_{0}\beta_{1}\leq 0$, then Y(s) can be realized by the network in Figure 5a with*

$$c=\frac{\alpha_{1}}{\beta_{1}},\;\;\;c^{\prime }=\frac{\alpha_{0}\beta_{1}-\alpha_{1}\beta_{0}}{\beta_{0}\beta_{1}},\;\;\;k=\frac{\alpha_{0}\beta_{1}-\alpha_{1}\beta_{0}}{\beta_{1}^{2}},$$

*and if $\alpha_{1}\beta_{0}-\alpha_{0}\beta_{1}>0$, then Y(s) can be realized by the network in Figure 5b with*

$$c=\frac{\alpha_{0}}{\beta_{0}},\;\;\;c^{\prime }=\frac{\alpha_{1}\beta_{0}-\alpha_{0}\beta_{1}}{\beta_{0}\beta_{1}},\;\;\;b=\frac{\alpha_{1}\beta_{0}-\alpha_{0}\beta_{1}}{\beta_{0}^{2}}.$$

Furthermore, the conclusions in Lemmas 3 and 4 will be applied to the asymptotic stability analysis in the proof of Propositions 1 and 2.

**Lemma** **3** ([46,47])**.** *Consider the following system as*

(2) $${\dot{x}}_{cl}=f\left(x_{cl},t\right),$$

*where $f:R^{n}\times \left[0,+\infty \right)\to R^{n}$, $x_{cl}\left(t\right)\in R^{n}$, and $x_{cl}=0$ is an equilibrium point. If there exists a continuously differentiable function $V:R^{n}\to R$ that simultaneously satisfies the following conditions:*

*1.*  *V(0)=0 and $V(x_{cl})>0$ for any $x_{cl}\neq 0$,*
*2.*  *$\dot{V}\left(x_{cl}\right)\leq -W\left(x_{cl}\right)\leq 0$,*
*3.*  *$W(x_{cl})≢0$ for any nonzero initial state, that is, $x_{cl}\left(t_{0}\right)\neq 0$,*
*4.*  *$V(x_{cl})\to \infty$ with $\left|\right|x_{cl}\left|\right|\to \infty$,*

*where $W:R^{n}\to R$ is a positive semi-definite continuous function, then the equilibrium point $x_{cl}=0$ of the system (or briefly called the system) in (2) is globally asymptotically stable.*

**Remark** **1.** *As stated in [46], when the system in Lemma 3 is a switched system, the continuously differentiable function $V:R^{n}\to R$ satisfying the conditions of Lemma 3 is named as the common Lyapunov function.*

**Lemma** **4** ([48])**.** *Consider the dynamic equation in the form of*

(3) $$M_{\sigma }\ddot{x}+\mathrm{Kx}=0,$$

*where $x\in R^{n}$, and the mass matrix $M_{\sigma }\in R^{n\times n}$ and the stiffness matrix $K\in R^{n\times n}$ are both positive definite symmetric. Let $\lambda_{1},\lambda_{2},\ldots ,\lambda_{n}$ be n roots of the characteristic equation $|K-\lambda M_{\sigma }|\;=\;0$, which means that $\lambda_{i}>0$ for i∈1,2,…,n, and let $U_{1},U_{2},\ldots ,U_{n}$ be the corresponding independent column eigenvectors. Then, the general solution x(t) of the dynamic equation in (3) can be expressed as*

x(t)=Uq(t),

*where $U=\left[U_{1}\;U_{2}\;\ldots \;U_{n}\right]\in R^{n\times n}$ and $q\in R^{n}$ satisfies*

$$q\left(t\right)=\Phi_{t}q\left(t_{0}\right)+\Psi_{t}\Omega^{-1}\dot{q}\left(t_{0}\right),$$

*with $\Phi_{t}=diag\left\{\cos\omega_{1}t,\;\cos\omega_{2}t,\;\ldots ,\;\cos\omega_{n}t\right\}$, $\Psi_{t}=diag\left\{\sin\omega_{1}t,\;\sin\omega_{2}t,\;\ldots ,\;\sin\omega_{n}t\right\}$, $\Omega =diag\{\omega_{1},\;\omega_{2},\;\ldots ,\;\omega_{n}\}$, and $\omega_{i}=\sqrt{\lambda_{i}}$ for i ∈{1,2,…,n}.*

## 3. Model Formulation

Regardless of any external disturbance, the *n*-DOF vibration control system to be investigated in this paper is shown in Figure 3, and consists of *n* masses, *n* springs, at most *n* dampers, at most *n* passive inerters, a semi-active switched inerter $b_{\mathrm{semi}}$, and a one-port linear time-invariant passive mechanical network whose admittance is Y(s). Here, $x_{i}$ for i∈{1,2,…,n} denotes the displacement of the *i*th mass, and $m_{i}>0$, $k_{i}>0$, $c_{i}\geq 0$, and $b_{i}\geq 0$ for i∈{1,2,…,n} denote the element values of the *i*th mass, spring, damper, and inerter, respectively.

The semi-active inerter $b_{\mathrm{semi}}$ is the semi-active controller that is installed between $m_{l-1}$ and $m_{l}$ for a certain l∈{1,2,…,n}, and its element value satisfies the following relative-velocity–relative-acceleration switching law as

(4) $$b_{\mathrm{semi}}\left(t\right)=\left\{\begin{array}{c} b_{a}>0\;\left({\dot{x}}_{l-1}-{\dot{x}}_{l}\right)\left({\ddot{x}}_{l-1}-{\ddot{x}}_{l}\right)\leq 0,\\ b_{b}>0\;\left({\dot{x}}_{l-1}-{\dot{x}}_{l}\right)\left({\ddot{x}}_{l-1}-{\ddot{x}}_{l}\right)>0, \end{array}\right.$$

and we assume that $x_{0}=0$. This switching law was proposed by [28] and can be implemented by the semi-active fluid inerter (see Figure 1).

The one-port linear time-invariant passive mechanical network, whose admittance is Y(s), acts as the passive controller that is installed between $m_{p-1}$ and $m_{p}$ for a certain p∈{1,2,…,n}. By Lemma 1, the admittance Y(s) of any passive mechanical controller is a positive-real function, and any positive-real admittance Y(s) is realizable as a one-port damper–spring–inerter network.

First, the dynamic equation of the *n*-DOF vibration system in Figure 3, excluding the semi-active inerter $b_{\mathrm{semi}}$ and the passive mechanical network Y(s), can be formulated as

(5) $$\left(M+B\right)\ddot{x}+C\dot{x}+Kx=T_{1}u_{\mathrm{semi}}+T_{2}u,$$

where $x={\left[x_{1}\;\;x_{2}\;\;\ldots \;\;x_{n}\right]}^{T}$,

$$M=\mathrm{diag}\{m_{1},m_{2},\ldots ,m_{n}\},$$

$$\begin{array}{c} B=\left[\begin{array}{ccccc} b_{1}+b_{2} & -b_{2} & \; & & \\ -b_{2} & b_{2}+b_{3} & -b_{3} & \; & \\ \; & \ddots & \ddots & \ddots & \\ \; & \; & -b_{n-1} & b_{n-1}+b_{n} & -b_{n}\\ \; & \; & & -b_{n} & b_{n} \end{array}\right], \end{array}$$

$$\begin{array}{c} C=\left[\begin{array}{ccccc} c_{1}+c_{2} & -c_{2} & \; & & \\ -c_{2} & c_{2}+c_{3} & -c_{3} & \; & \\ \; & \ddots & \ddots & \ddots & \\ \; & \; & -c_{n-1} & c_{n-1}+c_{n} & -c_{n}\\ \; & \; & & -c_{n} & c_{n} \end{array}\right], \end{array}$$

$$K=\left[\begin{array}{ccccc} k_{1}+k_{2} & -k_{2} & \; & & \\ -k_{2} & k_{2}+k_{3} & -k_{3} & \; & \\ \; & \ddots & \ddots & \ddots & \\ \; & \; & -k_{n-1} & k_{n-1}+k_{n} & -k_{n}\\ \; & \; & & -k_{n} & k_{n} \end{array}\right],$$

$$T_{1}=\left\{\begin{array}{c} {\left[\begin{array}{cc} 1 & 0_{1\times (n-1)} \end{array}\right]}^{T}\;l=1,\\ {\left[\begin{array}{cccc} 0_{1\times (l-2)} & -1 & 1 & 0_{1\times (n-l)} \end{array}\right]}^{T}\;l\geq 2, \end{array}\right.$$

and

$$T_{2}=\left\{\begin{array}{c} {\left[\begin{array}{cc} 1 & 0_{1\times (n-1)} \end{array}\right]}^{T}\;p=1,\\ {\left[\begin{array}{cccc} 0_{1\times (p-2)} & -1 & 1 & 0_{1\times (n-p)} \end{array}\right]}^{T}\;p\geq 2. \end{array}\right.$$

**Figure 6:**
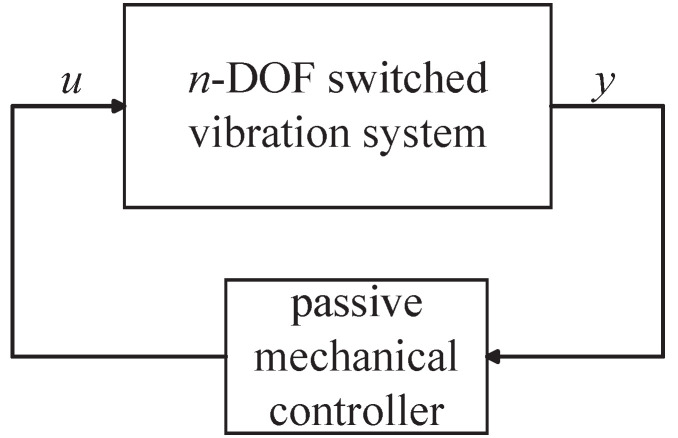
The control diagram of the *n*-DOF switched vibration system in Section 3.

Here, $u_{\mathrm{semi}}$ denotes the force provided by the semi-active inerter, and *u* denotes the force provided by the one-port passive mechanical network (see Figure 6).

Then, $u_{\mathrm{semi}}$ satisfies

(6) $$u_{\mathrm{semi}}=b_{\mathrm{semi}}\left({\ddot{x}}_{l-1}-{\ddot{x}}_{l}\right)=-b_{\mathrm{semi}}T_{1}^{T}\ddot{x},$$

where $b_{\mathrm{semi}}$ satisfies the switching law in (4). Substituting (6) into (5), we can derive the dynamic equation of the *n*-DOF switched vibration system containing the semi-active inerter as

(7) $$M_{\sigma }\ddot{x}+C\dot{x}+Kx=T_{2}u,$$

where $M_{\sigma }$ is related to $b_{\mathrm{semi}}$ and satisfies

(8) $$\begin{array}{c} M_{\sigma }\left(b_{\mathrm{semi}}\right)=M+B+b_{\mathrm{semi}}T_{1}T_{1}^{T}. \end{array}$$

For brevity, we define

$$M_{a}≜M_{\sigma }\left(b_{a}\right),\;\;\;M_{b}≜M_{\sigma }\left(b_{b}\right).$$

Furthermore, we can formulate the state-space equation of the switched vibration system (7) as

(9) $$\begin{array}{cc} {\dot{x}}_{s} & =A_{s}\left(t\right)x_{s}+B_{s}\left(t\right)u,\\ y & =C_{s}x_{s}, \end{array}$$

where $x_{s}={\left[x^{T}\;\;{\dot{x}}^{T}\right]}^{T}$,

$$\begin{array}{cc} A_{s}\left(t\right) & =\left\{\begin{array}{c} A_{s,1}\;{\dot{x}}^{T}T_{1}T_{1}^{T}\ddot{x}\leq 0,\\ A_{s,2}\;{\dot{x}}^{T}T_{1}T_{1}^{T}\ddot{x}>0, \end{array}\right.\\ B_{s}\left(t\right) & =\left\{\begin{array}{c} B_{s,1}\;{\dot{x}}^{T}T_{1}T_{1}^{T}\ddot{x}\leq 0,\\ B_{s,2}\;{\dot{x}}^{T}T_{1}T_{1}^{T}\ddot{x}>0, \end{array}\right.\\ C_{s} & =\left[\begin{array}{cc} 0_{1\times n} & -T_{2}^{T} \end{array}\right], \end{array}$$

with $A_{s,1}$, $A_{s,2}$, $B_{s,1}$, and $B_{s,2}$ satisfying

$$A_{s,1}=\left[\begin{array}{cc} 0_{n\times n} & I_{n\times n}\\ -M_{a}^{-1}K & -M_{a}^{-1}C \end{array}\right],\;\;\;A_{s,2}=\left[\begin{array}{cc} 0_{n\times n} & I_{n\times n}\\ -M_{b}^{-1}K & -M_{b}^{-1}C \end{array}\right],$$

$$B_{s,1}=\left[\begin{array}{c} 0_{n\times 1}\\ M_{a}^{-1}T_{2} \end{array}\right],\;\;\;B_{s,2}=\left[\begin{array}{c} 0_{n\times 1}\\ M_{b}^{-1}T_{2} \end{array}\right].$$

We note that the *n*-DOF vibration control system in Figure 3 can be regarded as the output-feedback control system whose diagram is shown in Figure 6. The plant to be controlled is the *n*-DOF switched vibration system (9) containing the semi-active inerter $b_{\mathrm{semi}}$, and the input and output of the plant are *u* and *y*, respectively. The passive controller whose admittance is Y(s) satisfies $\hat{u}=Y\left(s\right)\hat{y}$, where $\hat{u}$ and $\hat{y}$ are, respectively, the Laplace transforms of *u* and *y*. By Lemma 1, Y(s) must be positive real.

Considering any positive-real admittance Y(s) whose McMillan degree is *m*, we can express the minimal state-space realization of Y(s) as

(10) $$\begin{array}{cc} {\dot{x}}_{c} & =A_{c}x_{c}+B_{c}y,\\ u & =C_{c}x_{c}+D_{c}y, \end{array}$$

where $x_{c}\in R^{m}$, $A_{c}\in R^{m\times m}$, $B_{c}\in R^{m}$, $C_{c}^{T}\in R^{m}$, and $D_{c}\in R$.

Finally, combining (9) and (10) implies the state equation of the closed-loop vibration control system in Figure 3 is

(11) $${\dot{x}}_{cl}=A_{cl}\left(t\right)x_{cl}=\left\{\begin{array}{c} A_{cl,1}x_{cl}\;{\dot{x}}^{T}T_{1}T_{1}^{T}\ddot{x}\leq 0,\\ A_{cl,2}x_{cl}\;{\dot{x}}^{T}T_{1}T_{1}^{T}\ddot{x}>0, \end{array}\right.$$

where $x_{cl}={\left[x_{s}^{T}\;\;x_{c}^{T}\right]}^{T}$, and

$$A_{cl,1}=\left[\begin{array}{cc} A_{s,1}+B_{s,1}D_{c}C_{s} & B_{s,1}C_{c}\\ B_{c}C_{s} & A_{c} \end{array}\right],\;\;\;A_{cl,2}=\left[\begin{array}{cc} A_{s,2}+B_{s,2}D_{c}C_{s} & B_{s,2}C_{c}\\ B_{c}C_{s} & A_{c} \end{array}\right].$$

## 4. Stability Analysis

This section will investigate the global asymptotic stability in the sense of Lyapunov for the *n*-DOF vibration control system shown in Figure 3 whose state-space model has been formulated in Section 3. Specifically, the admittance Y(s) of the passive controller satisfies the following assumption.

**Assumption** **1.** *The admittance Y(s) is any first-order real-rational function as in (1), where $\alpha_{1}\geq 0$, $\alpha_{0}>0$, $\beta_{1}>0$, $\beta_{0}>0$, and $\alpha_{1}\beta_{0}-\alpha_{0}\beta_{1}\neq 0$.*

By Lemma 2, the first-order admittance Y(s) satisfying Assumption 1 must be positive real, and is realizable as a one-port three-element damper–spring–inerter network shown in Figure 5. Furthermore, the condition of $\alpha_{1}\beta_{0}-\alpha_{0}\beta_{1}\neq 0$ in Assumption 1 implies that the McMillan degree of Y(s) satisfies m=1, and one of the minimal state-space realizations in (10) can be determined to be

(12) $${\dot{x}}_{c}=A_{c}x_{c}+B_{c}y,\;\;\;u=C_{c}x_{c}+D_{c}y,$$

where $x_{c}\in R$, y∈R, u∈R, and

(13) $$A_{c}=-\frac{\beta_{0}}{\beta_{1}},\;\;\;B_{c}=1,\;\;\;C_{c}=\frac{\alpha_{0}\beta_{1}-\alpha_{1}\beta_{0}}{\beta_{1}^{2}},\;\;\;D_{c}=\frac{\alpha_{1}}{\beta_{1}}.$$

**Proposition** **1.** *Consider the n-DOF switched vibration control system in Figure 3 whose closed-loop state equation is (11), where $m_{i}>0$, $k_{i}>0$, $c_{i}\geq 0$, $b_{i}\geq 0$ for i∈{1,2,…,n}, l and p are independent given values satisfying l,p∈{1,2,…,n}, $b_{\mathrm{semi}}$ satisfies the switching law in (4), and the positive-real admittance Y(s) satisfies Assumption 1. If $b_{b}>b_{a}\geq 0$, the characteristic equation $|K-\lambda M_{a}|=0$ has no multiple root in λ, and the corresponding eigenvector matrix U satisfies $U_{l-1,j}\neq U_{l,j}$ or $U_{p-1,j}\neq U_{p,j}$ ($U_{i,j}$ denotes the (i,j)th entry of U and $U_{0,j}=0$) for any j∈{1,2,…,n}, then the closed-loop system (11) is globally asymptotically stable.*

**Proof.**  Define

$$b_{\alpha }≜\frac{b_{b}+b_{a}}{2},\;\;\;b_{\beta }≜\frac{b_{b}-b_{a}}{2},$$

which implies that $b_{\alpha }>0$ and $b_{\beta }>0$ because of $b_{b}>b_{a}>0$. Then, the switching law of $b_{\mathrm{semi}}$ in (4) can be equivalent to

(14) $$b_{\mathrm{semi}}=b_{\alpha }+b_{\beta }\mathrm{sgn}\left({\dot{x}}^{T}T_{1}T_{1}^{T}\ddot{x}\right),$$

where

$$\mathrm{sgn}\left({\dot{x}}^{T}T_{1}T_{1}^{T}\ddot{x}\right)=\left\{\begin{array}{c} -1\;{\dot{x}}^{T}T_{1}T_{1}^{T}\ddot{x}\leq 0,\\ 1\;{\dot{x}}^{T}T_{1}T_{1}^{T}\ddot{x}>0. \end{array}\right.$$

Choose the common Lyapunov function candidate $V:R^{2n+1}\to R$ as

(15) $$V\left(x_{cl}\right)=\frac{1}{2}x^{T}Kx+\frac{1}{2}{\dot{x}}^{T}\left(M+B+b_{\alpha }T_{1}T_{1}^{T}\right)\dot{x}+\frac{1}{2}\left|C_{c}\right|x_{c}^{2},$$

which implies that V(0)=0. Since it can be verified that K≻0, M≻0, B⪰0, $b_{\alpha }T_{1}T_{1}^{T}⪰0$, and $|C_{c}|>0$, we imply that $V(x_{cl})>0$ for any $x_{cl}\neq 0$. Moreover, it is obvious that $V(x_{cl})\to \infty$ with $∥x_{cl}∥\to \infty$. Taking the derivative of *V* in (15) along the trajectories of the system (11) implies

$$\begin{array}{cc} \dot{V}\left(x_{cl}\right)= & x^{T}K\dot{x}+{\dot{x}}^{T}\left(M+B+b_{\alpha }T_{1}T_{1}^{T}\right)\ddot{x}+\left|C_{c}\right|x_{c}{\dot{x}}_{c}\\ = & x^{T}K\dot{x}+{\dot{x}}^{T}\left(M+B+b_{\mathrm{semi}}T_{1}T_{1}^{T}\right)\ddot{x}-b_{\beta }\mathrm{sgn}\left({\dot{x}}^{T}T_{1}T_{1}^{T}\ddot{x}\right){\dot{x}}^{T}T_{1}T_{1}^{T}\ddot{x}+\left|C_{c}\right|x_{c}{\dot{x}}_{c}\\ = & x^{T}K\dot{x}+{\dot{x}}^{T}M_{\sigma }\ddot{x}-b_{\beta }|{\dot{x}}^{T}T_{1}T_{1}^{T}\ddot{x}\left|+\right|C_{c}|x_{c}{\dot{x}}_{c}\\ = & x^{T}K\dot{x}+{\dot{x}}^{T}\left(T_{2}u-C\dot{x}-Kx\right)-b_{\beta }|{\dot{x}}^{T}T_{1}T_{1}^{T}\ddot{x}\left|+\right|C_{c}|x_{c}{\dot{x}}_{c}\\ = & -{\dot{x}}^{T}C\dot{x}-b_{\beta }|{\dot{x}}^{T}T_{1}T_{1}^{T}\ddot{x}\left|-yu+\right|C_{c}|x_{c}{\dot{x}}_{c}\\ = & -{\dot{x}}^{T}C\dot{x}-b_{\beta }|{\dot{x}}^{T}T_{1}T_{1}^{T}\ddot{x}|+A_{c}|C_{c}|x_{c}^{2}-D_{c}y^{2}-(C_{c}-B_{c}|C_{c}\left|\right)x_{c}y\\ ≜ & -W(x_{cl}), \end{array}$$

where the second equality follows from (14), the third equality follows from (8), the fourth equality follows from (7), and the sixth equality follows from (12). If $\alpha_{1}\beta_{0}-\alpha_{0}\beta_{1}<0$, then it follows from (13) that

(16) $$W\left(x_{cl}\right)={\dot{x}}^{T}C\dot{x}+b_{\beta }\left|{\dot{x}}^{T}T_{1}T_{1}^{T}\ddot{x}\right|+\frac{\beta_{0}\left(\alpha_{0}\beta_{1}-\alpha_{1}\beta_{0}\right)}{\beta_{1}^{3}}x_{c}^{2}+\frac{\alpha_{1}}{\beta_{1}}y^{2}\geq 0.$$

If $\alpha_{1}\beta_{0}-\alpha_{0}\beta_{1}>0$, then it follows from (13) that

(17) $$W\left(x_{cl}\right)={\dot{x}}^{T}C\dot{x}+b_{\beta }\left|{\dot{x}}^{T}T_{1}T_{1}^{T}\ddot{x}\right|+\frac{\alpha_{1}\beta_{0}-\alpha_{0}\beta_{1}}{\beta_{1}\beta_{0}}{\left(y-\frac{\beta_{0}}{\beta_{1}}x_{c}\right)}^{2}+\frac{\alpha_{0}}{\beta_{0}}y^{2}\geq 0.$$

Now, it remains to prove that $W(x_{cl})≢0$ for any nonzero initial state $x_{cl}\left(t_{0}\right)$. Let us first discuss the general case when $\alpha_{1}>0$. Assume that $W(x_{cl})\equiv 0$. Then, it follows from (16) and (17) that ${\dot{x}}^{T}C\dot{x}\equiv 0$, ${\dot{x}}^{T}T_{1}T_{1}^{T}\ddot{x}\equiv 0$, $x_{c}\equiv 0$, and y≡0. Since ${\dot{x}}^{T}C\dot{x}=\sum_{i=1}^{n}c_{i}{\left({\dot{x}}_{i-1}-{\dot{x}}_{i}\right)}^{2}\equiv 0$ where it is assumed that ${\dot{x}}_{0}=0$, we can indicate that $c_{i}\left({\dot{x}}_{i-1}-{\dot{x}}_{i}\right)\equiv 0$, which further implies that $C\dot{x}\equiv 0$. Moreover, we imply from $x_{c}\equiv 0,y\equiv 0$, and (12) that u≡0. Moreover, since ${\dot{x}}^{T}T_{1}T_{1}^{T}\ddot{x}=\left({\dot{x}}_{l-1}-{\dot{x}}_{l}\right)\left({\ddot{x}}_{l-1}-{\ddot{x}}_{l}\right)\equiv 0$, it is only possible that $b_{\mathrm{semi}}=b_{a}$ by the switching law in (4). Then, the dynamic equation in (7) becomes

(18) $$M_{a}\ddot{x}+Kx=0.$$

Let $\lambda_{1},\lambda_{2},\ldots ,\lambda_{n}$ be the roots of the characteristic equation $|K-\lambda M_{a}|=0$ in λ, and let U be the corresponding eigenvector matrix. Since $M_{a}$ and K are both positive definite matrices, then $\lambda_{i}>0$ for i∈{1,2,…,n}. By Lemma 4, the solution of (18) can be expressed as

(19) $$x_{i}\left(t\right)=\sum_{j=1}^{n}U_{i,j}R_{j}\sin\left(\omega_{j}t+\varphi_{j}\right),$$

where $U_{i,j}$ is the (i,j)th entry of the eigenvector matrix U, the values of $R_{j}\in R$ and $\phi_{j}\in \left(-\pi /2,\pi /2\right)$ are determined by $x_{s}\left(t_{0}\right)$, and $\omega_{j}=\sqrt{\lambda_{j}}$. Recalling that ${\dot{x}}^{T}T_{1}T_{1}^{T}\ddot{x}=\left({\dot{x}}_{l-1}-{\dot{x}}_{l}\right)\left({\ddot{x}}_{l-1}-{\ddot{x}}_{l}\right)\equiv 0$, we can derive that

$${\dot{x}}_{l-1}-{\dot{x}}_{l}=\sum_{j=1}^{n}\left(U_{l-1,j}-U_{l,j}\right)\omega_{j}R_{j}\cos\left(\omega_{j}t+\varphi_{j}\right)\equiv 0,$$

or

$${\ddot{x}}_{l-1}-{\ddot{x}}_{l}=-\sum_{j=1}^{n}\left(U_{l-1,j}-U_{l,j}\right)\omega_{j}^{2}R_{j}\sin\left(\omega_{j}t+\varphi_{j}\right)\equiv 0.$$

Moreover, it is implied from $y={\dot{x}}_{p-1}-{\dot{x}}_{p}\equiv 0$ that

$${\dot{x}}_{p-1}-{\dot{x}}_{p}=\sum_{j=1}^{n}\left(U_{p-1,j}-U_{p,j}\right)\omega_{j}R_{j}\cos\left(\omega_{j}t+\varphi_{j}\right)\equiv 0.$$

Furthermore, we can imply that $R_{j}\equiv 0$ for j∈{1,2,…,n} since the characteristic equation $|K-\lambda M_{a}|=0$ has no multiple root in λ, and the corresponding eigenvector matrix U satisfies $U_{l-1,j}\neq U_{l,j}$ or $U_{p-1,j}\neq U_{p,j}$ for j∈{1,2,…,n}. Then, we can directly imply from (19) that $x_{i}\equiv 0$ for i∈{1,2,…,n}, which means that $x_{s}\equiv 0$. Recalling that $x_{c}\equiv 0$, we conclude that $x_{cl}={\left[x_{s}^{T}\;\;x_{c}^{T}\right]}^{T}\equiv 0$. By the method of contradiction, it is implied that $W(x_{cl})≢0$ for any $x_{cl}\left(t_{0}\right)\neq 0$. In addition, for the specific case when $\alpha_{1}=0$, we can similarly prove that $W(x_{cl})≢0$ for any $x_{cl}\left(t_{0}\right)\neq 0$.

As a conclusion, by Lemma 3, the closed-loop vibration control system (11) is globally asymptotically stable. □

**Remark** **2.** *When $\alpha_{1}\beta_{0}-\alpha_{0}\beta_{1}=0$, Y(s) in (1) can be expressed as Y(s)=c where c>0. Then, the state equation of the closed-loop vibration control system can be obtained as*

(20) $${\dot{x}}_{s}=A_{cl}\left(t\right)x_{s}=\left(A_{s}\left(t\right)+cB_{s}\left(t\right)C_{s}\right)x_{s}.$$

*If the condition of Proposition 1 holds, then the closed-loop system (20) is also globally asymptotically stable. The proof is similar to that of Proposition 1.*

**Figure 7:**
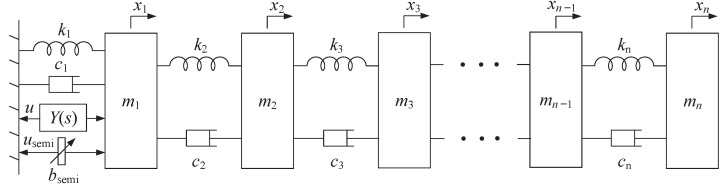
The specific *n*-DOF vibration control system.

Similarly, we will discuss the global asymptotic stability problem for the specific case of Figure 3 when $b_{i}=0$ for i∈{1,2,…,n}, that is, B=0, and both of $b_{\mathrm{semi}}$ and Y(s) are installed between the base and $m_{1}$ (that is l=p=1). This specific case is equivalently shown in Figure 7, and the following proposition presents a sufficient condition for the global asymptotic stability of this system, where the condition is more general than that of Proposition 1.

**Proposition** **2.** *Consider the n-DOF switched vibration control system in Figure 3 whose closed-loop state equation is (11), where $m_{i}>0$, $k_{i}>0$, $c_{i}\geq 0$, $b_{i}=0$ for i∈{1,2,…,n}, $b_{\mathrm{semi}}$ satisfies the switching law in (4), the positive-real admittance Y(s) satisfies Assumption 1, and both of $b_{\mathrm{semi}}$ and Y(s) are installed between the base and $m_{1}$ (that is, l=p=1). If $b_{b}\geq b_{a}\geq 0$, then the closed-loop system (11) is globally asymptotically stable.*

**Proof.**  Choose the common Lyapunov function candidate $V:R^{2n+1}\to R$ as (15), where B=0 and $T_{1}={\left[1\;\;0_{1\times (n-1)}\right]}^{T}$.

Similarly to the proof of Proposition 1, taking the derivative of *V* in (15) along the trajectories of the system (11), it remains to prove that $W(x_{cl})≢0$ for any nonzero initial state $x_{cl}\left(t_{0}\right)$. Let us first discuss the general case when $\alpha_{1}>0$ and $b_{\beta }>0$. Assume that $W(x_{cl})\equiv 0$. The dynamic equation in (7) can be simplified to (18). Since ${\dot{x}}^{T}T_{1}T_{1}^{T}\ddot{x}={\dot{x}}_{1}{\ddot{x}}_{1}\equiv 0$, which means that ${\dot{x}}_{1}\equiv 0$ or ${\ddot{x}}_{1}\equiv 0$, we can derive that $x_{1}\equiv h_{1}$ or $x_{1}\equiv h_{2}t+h_{3}$, where $h_{1}$, $h_{2}$, and $h_{3}\in R$. Then, it follows from (19) that $h_{1}=h_{2}=h_{3}\equiv 0$, implying $x_{1}\equiv 0$. By substituting $x_{1}\equiv 0$ into (18), we further imply that $x_{s}\equiv 0$. Recalling that $x_{c}\equiv 0$, we conclude that $x_{cl}={\left[x_{s}^{T}\;\;x_{c}^{T}\right]}^{T}\equiv 0$. By the method of contradiction, it is implied that $W(x_{cl})≢0$ for any $x_{cl}\left(t_{0}\right)\neq 0$. In addition, for the specific case when $\alpha_{1}=0$ or $b_{\beta }=0$, we can similarly prove that $W(x_{cl})≢0$ for any $x_{cl}\left(t_{0}\right)\neq 0$.

As a conclusion, by Lemma 3, the closed-loop vibration control system (11) is globally asymptotically stable. □

## 5. Optimization Design

This section will further investigate the optimization design problems of two *n*-DOF vibration control systems, including a quarter-car suspension control system (n=2) and a three-storey building vibration control system (n=3), respectively. In the design process, the parameters of $b_{\mathrm{semi}}$ ($b_{a}$ and $b_{b}$) are chosen to satisfy the stability analysis results derived in Section 4 to guarantee the systems are globally asymptotically stable, and the optimal parameters of Y(s) are determined by optimizing the system performances. Then, the optimal positive-real admittance can be physically realized as a one-port damper–spring–inerter network by the theory of passive network synthesis.

### 5.1. Quarter-Car Suspension Control System

**Figure 8:**
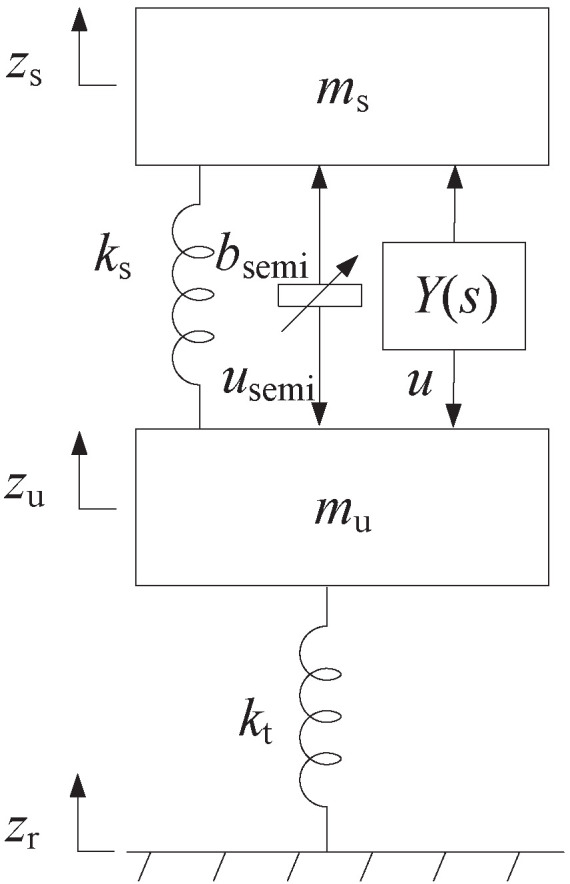
The quarter-car suspension control system in Section 5.1.

The quarter-car suspension control system shown in Figure 8 consists of a sprung mass $m_{s}$, an unsprung mass $m_{u}$, and a tyre whose spring stiffness is $k_{t}$. Moreover, the semi-active inerter $b_{\mathrm{semi}}$ and the passive mechanical controller Y(s) are installed between $m_{s}$ and $m_{u}$, where $b_{\mathrm{semi}}$ satisfies the switching law in (4), and Y(s) is the first-order positive-real admittance satisfying Assumption 1. The suspension control system can be regarded as the *n*-DOF vibration system in Figure 3 where n=l=p=2, $b_{1}=b_{2}=0$, and $c_{1}=c_{2}=0$, and the road disturbance $z_{r}$ is the external input of the system.

The dynamic equation of the suspension control system in Figure 8 is

$$M\ddot{x}+Kx=T_{1}u_{\mathrm{semi}}+T_{2}u+T_{3}z_{r},$$

where $x={\left[z_{u}\;z_{s}\right]}^{T}$, $u_{\mathrm{semi}}$ satisfying (6) is the force provided by the semi-active inerter, *u* satisfying (12) and (13) is the force provided by the passive controller, $z_{r}$ is the external input, and

$$\begin{array}{c} M=\left[\begin{array}{cc} m_{u} & 0\\ 0 & m_{s} \end{array}\right],\;K=\left[\begin{array}{cc} k_{t}+k_{s} & -k_{s}\\ -k_{s} & k_{s} \end{array}\right],\\ T_{1}=\left[\begin{array}{c} -1\\ 1 \end{array}\right],\;T_{2}=\left[\begin{array}{c} -1\\ 1 \end{array}\right],\;T_{3}=\left[\begin{array}{c} k_{t}\\ 0 \end{array}\right]. \end{array}$$

Then, following the procedure in Section 3, we can formulate the closed-loop state equation of the switched suspension control system as

(21) $${\dot{x}}_{cl}=A_{cl}\left(t\right)x_{cl}+B_{cl}\left(t\right)z_{r}=\left\{\begin{array}{c} A_{cl,1}x_{cl}+B_{cl,1}z_{r}\;{\dot{x}}^{T}T_{1}T_{1}^{T}\ddot{x}\leq 0,\\ A_{cl,2}x_{cl}+B_{cl,2}z_{r}\;{\dot{x}}^{T}T_{1}T_{1}^{T}\ddot{x}>0, \end{array}\right.$$

where $x_{cl}={\left[z_{u}\;z_{s}\;{\dot{z}}_{u}\;{\dot{z}}_{s}\;x_{c}\right]}^{T}$. It is well-known that introducing the external input $z_{r}$ does not alter the system stability in the sense of Lyapunov. By choosing $z_{s}$ and $k_{t}\left(z_{u}-z_{r}\right)$ as the external outputs, we can obtain the output equation as

(22) $$z=\left[\begin{array}{c} z_{s}\\ k_{t}\left(z_{r}-z_{u}\right) \end{array}\right]=C_{cl}x_{cl}+D_{cl}z_{r},$$

where

$$C_{cl}=\left[\begin{array}{ccccc} 0 & 1 & 0 & 0 & 0\\ -k_{t} & 0 & 0 & 0 & 0 \end{array}\right],\;\;\;D_{cl}=\left[\begin{array}{c} 0\\ k_{t} \end{array}\right].$$

For two subsystems of the switched system in (21) and (22), we can determine the transfer functions from $z_{r}$ to $z_{s}$ as

$$\begin{array}{cc} T_{{\hat{z}}_{r}\to {\hat{z}}_{s}}^{\left(1\right)} & =D_{cl}\left(1,:\right)+C_{cl}\left(1,:\right){\left(sI-A_{cl,1}\right)}^{-1}B_{cl,1},\\ T_{{\hat{z}}_{r}\to {\hat{z}}_{s}}^{\left(2\right)} & =D_{cl}\left(1,:\right)+C_{cl}\left(1,:\right){\left(sI-A_{cl,2}\right)}^{-1}B_{cl,2}, \end{array}$$

and the transfer functions from $z_{r}$ to $k_{t}\left(z_{r}-z_{u}\right)$ as

$$\begin{array}{cc} T_{{\hat{z}}_{r}\to k_{t}\left({\hat{z}}_{r}-{\hat{z}}_{u}\right)}^{\left(1\right)} & =D_{cl}\left(2,:\right)+C_{cl}\left(2,:\right){\left(sI-A_{cl,1}\right)}^{-1}B_{cl,1},\\ T_{{\hat{z}}_{r}\to k_{t}\left({\hat{z}}_{r}-{\hat{z}}_{u}\right)}^{\left(2\right)} & =D_{cl}\left(2,:\right)+C_{cl}\left(2,:\right){\left(sI-A_{cl,2}\right)}^{-1}B_{cl,2}, \end{array}$$

where $C_{cl}\left(i,:\right)$ and $D_{cl}\left(i,:\right)$ for i∈{1,2} represent the *i*th rows of $C_{cl}$ and $D_{cl}$, respectively.

Based on [49], the ride comfort performances corresponding to the two subsystems are defined as

$$\begin{array}{cc} J_{1,1} & =2\pi {\left(V\kappa \right)}^{1/2}{∥sT_{{\hat{z}}_{r}\to {\hat{z}}_{s}}^{\left(1\right)}∥}_{2},\\ J_{1,2} & =2\pi {\left(V\kappa \right)}^{1/2}{∥sT_{{\hat{z}}_{r}\to {\hat{z}}_{s}}^{\left(2\right)}∥}_{2}, \end{array}$$

and the road holding performances corresponding to the two subsystems are defined as

$$\begin{array}{c} J_{3,1}=2\pi {\left(V\kappa \right)}^{1/2}{∥\frac{1}{s}T_{{\hat{z}}_{r}\to k_{t}\left({\hat{z}}_{u}-{\hat{z}}_{r}\right)}^{\left(1\right)}∥}_{2},\\ J_{3,2}=2\pi {\left(V\kappa \right)}^{1/2}{∥\frac{1}{s}T_{{\hat{z}}_{r}\to k_{t}\left({\hat{z}}_{u}-{\hat{z}}_{r}\right)}^{\left(2\right)}∥}_{2}, \end{array}$$

where *V* denotes the vehicle speed, κ denotes the road roughness parameter, and $\left|\right|\cdot {\left|\right|}_{2}$ denotes the $H_{2}$ norm.

Combining the performances of two subsystems, we can define the ride comfort performance $J_{1}$ of the switched system as

$$J_{1}=W_{1}J_{1,1}+\left(1-W_{1}\right)J_{1,2},$$

and the road holding performance $J_{3}$ of the switched system as

$$J_{3}=W_{3}J_{3,1}+\left(1-W_{3}\right)J_{3,2},$$

where $W_{1}\in \left[0,1\right]$ and $W_{3}\in \left[0,1\right]$ are weighting factors.

Then, we simultaneously consider the above two performance indexes in the suspension system design. After determining the parameter values of $b_{\mathrm{semi}}$ ($b_{a}$ and $b_{b}$) satisfying Proposition 1, the parameter values of Y(s) can be optimized by solving the following optimization problem:

(23) $$\begin{array}{cc} \; & \min_{Y(s)}\;\;J=\rho \frac{J_{1}}{J_{1,0}}+\left(1-\rho \right)\frac{J_{3}}{J_{3,0}}\\ \; & s.t.\;\;Y(s)\;\mathrm{satisfies}\;\mathrm{Assumption}\;1, \end{array}$$

where the values of $J_{1,0}$, $J_{3,0}$, $W_{1}$, $W_{3}$, ρ, and $k_{s}$ are fixed. The combined performance index *J*, which includes the ride comfort and the tire holding performances, will be made optimal by solving the optimization problem (23).

Let the parameters of the system be taken from those in [49], where $m_{u}=35$ kg, $m_{s}=250$ kg, $k_{t}=150$ kN/m, $\kappa =5\times 10^{-7}$ m^3^ cycle^−1^, and V=25 m/s. Then, let $J_{1,0}=1.78$, $J_{3,0}=518.85$, $W_{1}=0.5$, $W_{3}=0.5$, ρ=0.4, and $k_{s}=80$ kN/m. According to the condition of Proposition 1, we can set the parameter values of the switched inerter $b_{\mathrm{semi}}$ to be $b_{a}=0$ and $b_{b}=25$ kg ($\lambda_{1}=205.11$, $\lambda_{2}=6686.32$, $U_{1,1}=-0.0225$, $U_{2,1}=-0.0627$, $U_{1,2}=-0.1675$, $U_{2,2}=0.0084$, and $b_{b}>b_{a}$), in order to guarantee the system is globally asymptotically stable. By solving the optimization problem in (23), we can determine the optimal values of Y(s) in (1) to be

$$\alpha_{1}=4.006\times 10^{3},\;\;\alpha_{0}=2.443\times 10^{-10},\;\;\beta_{1}=1,\;\;\beta_{0}=10.046\;.$$

By Lemma 2, Y(s) can be realized by the one-port three-element mechanical network in Figure 5b with the element values satisfying (it is noted that the value of *c* is close to zero, which means that this damper can be approximately open-circuited)

$$c=2.432\times 10^{-11}\;\mathrm{Ns}/m,\;\;c^{\prime }=4.006\times 10^{3}\;\mathrm{Ns}/m,\;\;b=3.988\times 10^{2}\;\mathrm{kg}.$$

For comparisons, we can similarly solve the optimization problems for the case when the suspension controllers include a semi-active inerter $b_{\mathrm{semi}}$ and a damper $c_{s}$ and for the case when the suspension controller is only a damper $c_{p}$. Using the similar optimization methods, we can determine the optimal values of $c_{s}$ and $c_{p}$ to be 3411.3 Ns/m and 3339.6 Ns/m, respectively. The optimal values of *J* for the above three cases are shown in the first row of Table 1. We can observe that the control strategy in this paper, which simultaneously applies a semi-active inerter and a passive mechanical network whose admittance is a first-order positive-real admittance, can outperform two other simpler mechanical control cases and provide 9.49% and 10.65% performance improvements, respectively.

**Figure 9:**
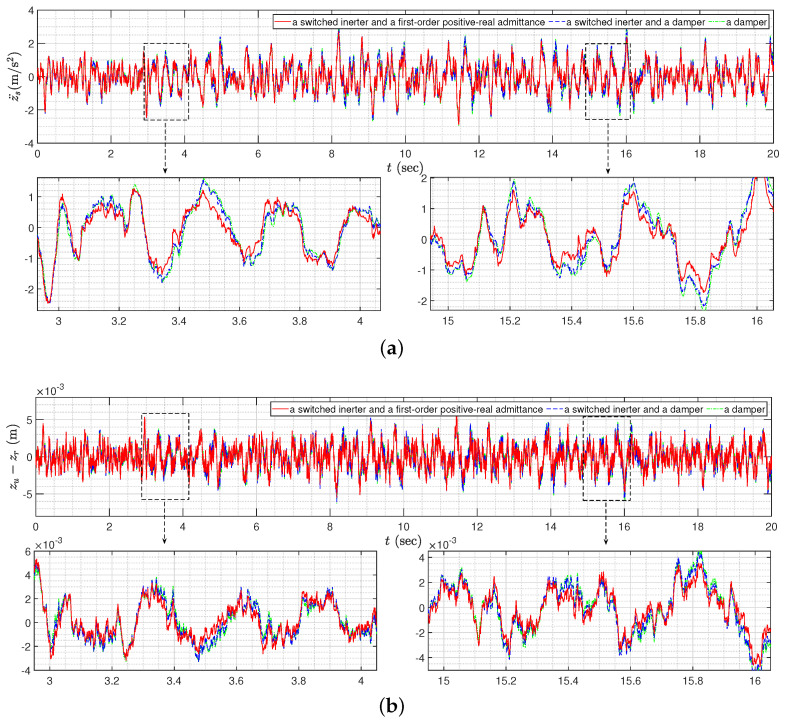
The time-domain responses of the quarter-car suspension control systems: (**a**) the sprung mass acceleration ${\ddot{z}}_{s}$; (**b**) the tire deflection $z_{u}-z_{r}$.

Next, we will discuss the time-domain responses to validate the optimization design results. According to [26], the road profile for the time-domain simulation can be described as

$${\dot{z}}_{r}\left(t\right)=-\alpha Vz_{r}\left(t\right)+w\left(t\right),$$

where w(t) is the white noise with the spectral density $\Psi_{w}=2\alpha V\sigma^{2}$, and V= 25 m/s is the vehicle speed. Let the values of the parameters α and σ satisfy 0.127 rad/m and $4\times 10^{-3}$ m (road class *B* in [26]). Then, the time-domain responses for the sprung mass acceleration ${\ddot{z}}_{s}$ and the tire deflection $z_{u}-z_{r}$ are shown in Figure 9, and the corresponding RMS (root-mean-square) values are presented in Table 1. It is noted that the RMS values of the responses for the control strategy in this paper are obviously smaller than two other cases, where the degradation percentages are 13.13% and 18.25% for the RMS of ${\ddot{z}}_{s}$ and are 9.79% and 10.62% for the RMS of $z_{u}-z_{r}$, respectively. The results validate that the control strategy in this paper can provide the best performances when suppressing the external disturbances.

**Figure 10:**
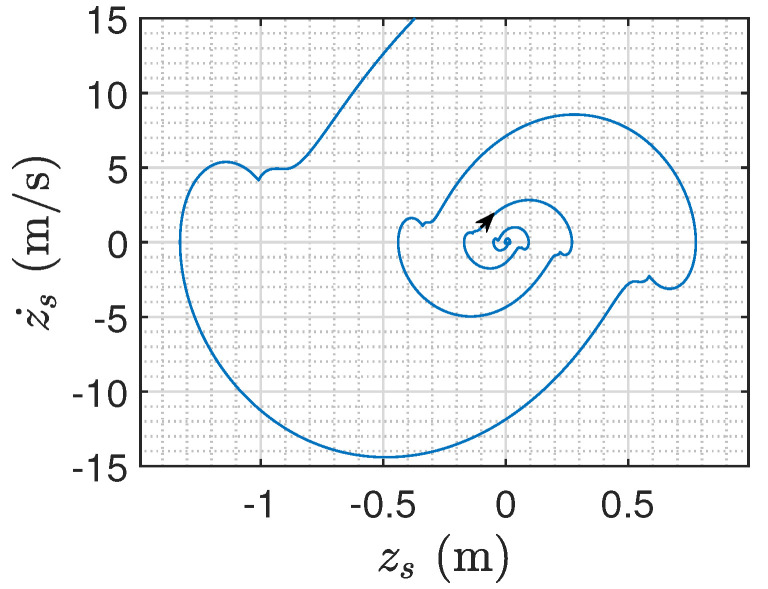
The divergent phase trajectory of the sprung mass $m_{s}$ when $b_{a}=2b_{b}$ (the direction of the trajectory is indicated by the arrow).

In addition, when the semi-active inerter satisfies $b_{a}=2b_{b}=50$ kg, contradicting the condition of Proposition 1, we can verify that the system is not globally asymptotically stable (see Figure 10).

### 5.2. Three-Storey Building Vibration Control System

**Figure 11:**
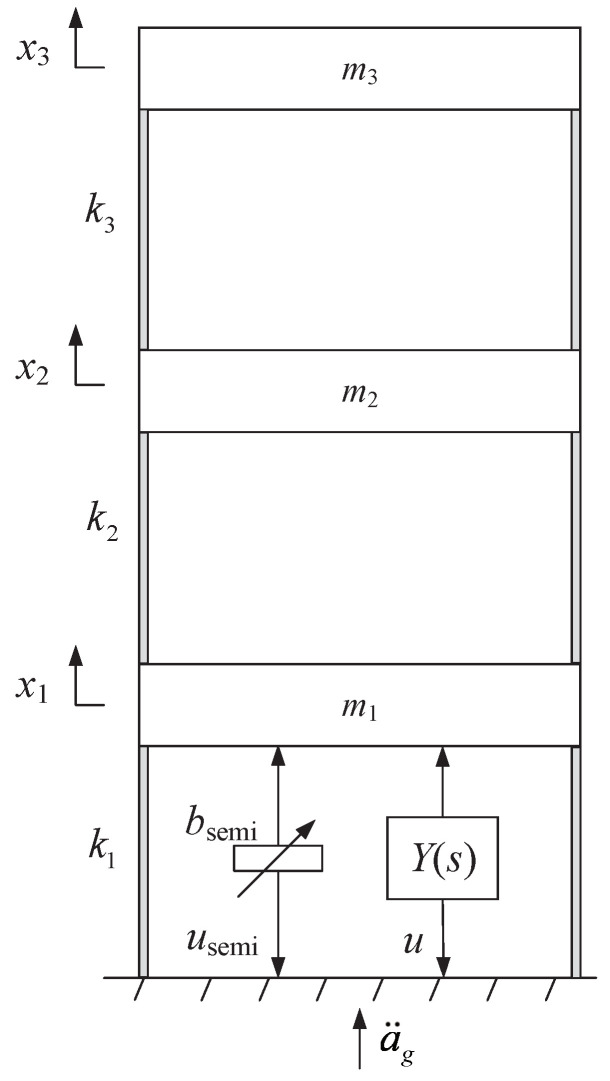
The three-storey building vibration control system in Section 5.2.

The three-storey building vibration control system is shown in Figure 11 (see [23,50]), where $x_{i}$ for i∈{1,2,3} denotes the relative displacement of the *i*th storey to the ground, ${\ddot{a}}_{g}$ denotes the acceleration of the ground, and the semi-active inerter $b_{\mathrm{semi}}$ and the passive controller Y(s) are installed between the ground and the first floor. The three-storey building vibration control system can be regarded as the *n*-DOF vibration system in Figure 3 where n=3, l=p=1, $m_{1}=m_{2}=m_{3}=1000$ kg, $k_{1}=k_{2}=k_{3}=1500$ kN/s, $b_{1}=b_{2}=b_{3}=0$, $c_{1}=c_{2}=c_{3}=0$, and the ground acceleration ${\ddot{a}}_{g}$ is the external input of the system.

The dynamic equation of the three-storey building vibration control system is

$$M\ddot{x}+Kx=T_{1}u_{\mathrm{semi}}+T_{2}u+T_{3}{\ddot{a}}_{g},$$

where $x={\left[x_{1}\;x_{2}\;x_{3}\right]}^{T}$, $u_{\mathrm{semi}}$ satisfying (6) is the force provided by the semi-active inerter, *u* satisfying (12) and (13) is the force provided by the passive controller, ${\ddot{a}}_{g}$ is the external input, and

$$\begin{array}{cc} M= & \left[\begin{array}{ccc} m_{1} & 0 & 0\\ 0 & m_{2} & 0\\ 0 & 0 & m_{3} \end{array}\right],\;K=\left[\begin{array}{ccc} k_{1}+k_{2} & -k_{2} & 0\\ -k_{2} & k_{2}+k_{3} & -k_{3}\\ 0 & -k_{3} & k_{3} \end{array}\right],\\ \; & T_{1}=\left[\begin{array}{c} 1\\ 0\\ 0 \end{array}\right],\;T_{2}=\left[\begin{array}{c} 1\\ 0\\ 0 \end{array}\right],\;T_{3}=-\left[\begin{array}{c} m_{1}\\ m_{2}\\ m_{3} \end{array}\right]. \end{array}$$

Then, following the procedure in Section 3, we can formulate the closed-loop state equation of the switched three-storey building control system as

(24) $${\dot{x}}_{cl}=A_{cl}\left(t\right)x_{cl}+B_{cl}\left(t\right){\ddot{a}}_{g}=\left\{\begin{array}{c} A_{cl,1}x_{cl}+B_{cl,1}{\ddot{a}}_{g}\;{\dot{x}}^{T}T_{1}T_{1}^{T}\ddot{x}\leq 0,\\ A_{cl,2}x_{cl}+B_{cl,2}{\ddot{a}}_{g}\;{\dot{x}}^{T}T_{1}T_{1}^{T}\ddot{x}>0, \end{array}\right.$$

where $x_{cl}={\left[x_{1}\;x_{2}\;x_{3}\;{\dot{x}}_{1}\;{\dot{x}}_{2}\;{\dot{x}}_{3}\;x_{c}\right]}^{T}$. By choosing $z_{1}=x_{1}$, $z_{2}=x_{2}-x_{1}$, and $z_{3}=x_{3}-x_{2}$ as the external outputs, we can obtain the output equation as

(25) $$z=\left[\begin{array}{c} x_{1}\\ x_{2}-x_{1}\\ x_{3}-x_{2} \end{array}\right]=C_{cl}x_{cl},$$

where

$$C_{cl}=\left[\begin{array}{ccccccc} 1 & 0 & 0 & 0 & 0 & 0 & 0\\ -1 & 1 & 0 & 0 & 0 & 0 & 0\\ 0 & -1 & 1 & 0 & 0 & 0 & 0 \end{array}\right].$$

For two subsystems of the switched system in (24) and (25), we can determine the transfer functions from ${\ddot{a}}_{g}$ to $z_{i}$ as

$$\begin{array}{cc} T_{s^{2}{\hat{a}}_{g}\to {\hat{z}}_{i}}^{\left(1\right)} & =C_{cl}\left(i,:\right){\left(sI-A_{cl,1}\right)}^{-1}B_{cl,1},\;\;i\in \left\{1,2,3\right\},\\ T_{s^{2}{\hat{a}}_{g}\to {\hat{z}}_{i}}^{\left(2\right)} & =C_{cl}\left(i,:\right){\left(sI-A_{cl,2}\right)}^{-1}B_{cl,2},\;\;i\in \left\{1,2,3\right\}, \end{array}$$

where $C_{cl}\left(i,:\right)$ denotes the *i*th row of $C_{cl}$.

Based on [51], the performances for the two subsystems can be defined as

$$\begin{array}{cc} J_{1} & =\max\left({∥T_{s^{2}{\hat{a}}_{g}\to {\hat{z}}_{i}}^{\left(1\right)}∥}_{\infty }\right),\;\;\;i\in \left\{1,2,3\right\},\\ J_{2} & =\max\left({∥T_{s^{2}{\hat{a}}_{g}\to {\hat{z}}_{i}}^{\left(2\right)}∥}_{\infty }\right),\;\;\;i\in \left\{1,2,3\right\}, \end{array}$$

where ${∥\cdot ∥}_{\infty }$ denotes the $H_{\infty }$ norm.

After determining the parameter values of $b_{\mathrm{semi}}$ ($b_{a}$ and $b_{b}$) satisfying the condition of Proposition 2, the parameter values of Y(s) can be optimized by solving the the following optimization problem:

(26) $$\begin{array}{cc} \; & \min_{Y(s)}\;\;J=\max\left(J_{1},J_{2}\right)\\ \; & s.t.\;\;Y(s)\;\mathrm{satisfies}\;\mathrm{Assumption}\;1. \end{array}$$

By solving the optimization problem in (26), we can determine the optimal values of Y(s) in (1) to be

$$\alpha_{1}=1.403\times 10^{5},\;\;\alpha_{0}=2.386\times 10^{6},\;\;\beta_{1}=1,\;\;\beta_{0}=29.549\;.$$

By Lemma 2, Y(s) can be realized by the one-port three-element mechanical network in Figure 5b with the element values satisfying

$$c=8.075\times 10^{4}\;\mathrm{Ns}/m,\;\;c^{\prime }=5.953\times 10^{4}\;\mathrm{Ns}/m,\;\;b=2.015\times 10^{3}\;\mathrm{kg}.$$

For comparison, we can similarly solve the optimization problems for the case when the building controllers include a semi-active inerter $b_{\mathrm{semi}}$ and a damper $c_{s}$ and for the case when the building controller is only a damper $c_{p}$. Using the similar optimization methods, we can determine the optimal values of $c_{s}$ and $c_{p}$ to be 91,329.6 Ns/m and 94,584.7 Ns/m, respectively. The optimal values of *J* for the above three cases are shown in the first row of Table 2. We note that compared with two other simpler control cases, the control strategy in this paper can outperform them and provide 26.49% and 40.20% performance improvements, respectively.

**Figure 12:**
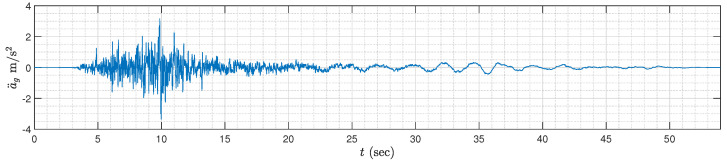
The time history of the Kobe earthquake.

**Figure 13:**
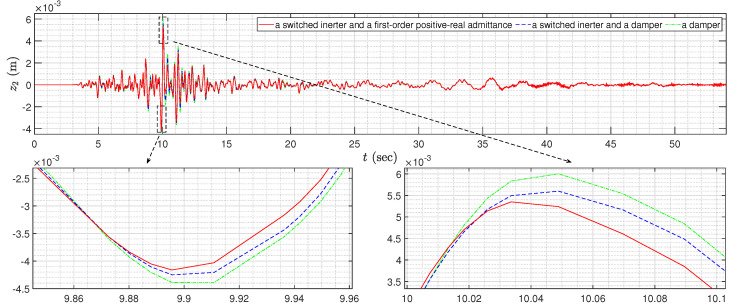
The time-domain responses (with 5% structural damping) of the three-storey building control systems.

To further verify the optimization design results, we will discuss the time-domain response under the Kobe earthquake record (see Figure 12), which took place in Japan on 16 January 1995. By introducing 5% structural damping (referred to [52]), the time-domain responses of $z_{2}=x_{2}-x_{1}$, which is the inter-drift between the first and second floor, is shown in Figure 13, and the corresponding maximum values of $|z_{2}|$ and the RMS (root-mean-square) value of $z_{2}$ for the above three cases are presented in Table 2. It is clear that compared with two other simpler cases, the control strategy in this paper provides the smallest maximum values of $|z_{i}|$ for i∈{1,2,3}, which, for instance, can provide 4.65% and 12.19% performance improvements on the values of $z_{2}$. Moreover, although the RMS value $z_{1}$ is slightly larger, the RMS values of $z_{2}$ and $z_{3}$ for the control strategy in this paper are obviously smaller than the other two cases. The above results validate that the control strategy in this paper, which simultaneously applies a semi-active inerter and a first-order positive-real admittance, can provide the best performances when suppressing the external disturbances.

**Figure 14:**
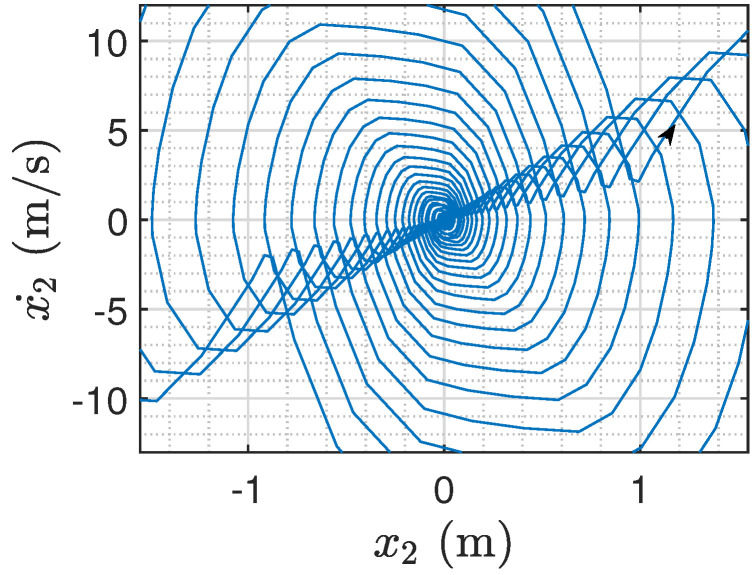
The divergent phase trajectory of the second floor mass $m_{2}$ when $b_{a}=50b_{b}$ (the direction of the trajectory is indicated by the arrow).

In addition, when the semi-active inerter satisfies $b_{a}=50b_{b}=50000$ kg, which contradicts the condition of Proposition 2, we can verify that the system is not globally asymptotically stable (see Figure 14).

## 6. Conclusions

This paper has conducted the stability analysis and design of a general *n*-DOF mass-chain vibration system that simultaneously utilizes a semi-active switched inerter and a passive mechanical network with a first-order positive-real admittance, which are, respectively, semi-active and passive mechanical controllers.

- Based on Lyapunov’s stability theory, a sufficient condition for the global asymptotic stability of a general *n*-DOF mass-chain vibration system, whose semi-active and passive controllers can be installed between any two adjacent masses, has been derived. Similarly, we have obtained a sufficient condition for a specific *n*-DOF mass-chain vibration system to be globally asymptotically stable.
- The stability conditions derived in this paper have been applied to the optimization designs of a vehicle suspension control system and a building vibration control system, respectively. The results reveal that simultaneously applying semi-active and passive mechanical controllers to vibration systems can clearly enhance system performances compared with the conventional semi-active or passive mechanical control methods.
- The research in this paper can provide important guidance for further investigation of the stability problems of more general systems, which is motivated by the designs of many vibration systems in practice.

## Figures and Tables

**Table 1:** The performance comparisons among the three cases for the quarter-car suspension control systems, which are the case when the controllers include a semi-active inerter and a first-order positive-real admittance (this paper), the case when the controllers include a semi-active inerter and a damper, and the case when the controller is only a damper.

Performance	A Switched Inerter and a First-Order Positive-Real Admittance (This Paper)	A Switched Inerter and a Damper	A Damper
*J*	1.007	1.102(−9.49%)	1.114(−10.65%)
RMS ${\ddot{z}}_{s}$ (m/$s^{2}$)	0.785	0.887(−13.13%)	0.928(−18.25%)
RMS $z_{u}-z_{r}$ (mm)	1.523	1.675(−9.79%)	1.687(−10.62%)

**Table 2:** The performance comparisons among the three cases for the three-storey building vibration control systems, which are the case when the controllers include a semi-active inerter and a first-order positive-real admittance (this paper), the case when the controllers include a semi-active inerter and a damper, and the case when the controller is only a damper.

Performance	A Switched Inerter and a First-Order Positive-Real Admittance (This Paper)	A Switched Inerter and a Damper	A Damper
*J* ($\times 10^{-3}$)	3.074	3.888(−26.49%)	4.309(−40.20%)
max $|z_{1}|$ (mm)	4.699	4.788(−1.88%)	4.794(−2.02%)
max $|z_{2}|$ (mm)	5.350	5.599(−4.65%)	6.003(−12.19%)
max $|z_{3}|$ (mm)	3.462	3.592(−3.76%)	3.792(−9.54%)
RMS $z_{1}$ (mm)	0.640	0.636(+0.49%)	0.634(+0.86%)
RMS $z_{2}$ (mm)	0.666	0.706(−5.90%)	0.752(−12.87%)
RMS $z_{3}$ (mm)	0.390	0.407(−4.42%)	0.434(−11.16%)

## Data Availability

Data are contained within the article.
